# Real‐world evidence of systemic treatment practices for biliary tract cancer in Japan: Results of a database study

**DOI:** 10.1002/jhbp.1418

**Published:** 2024-05-27

**Authors:** Makoto Ueno, Sachiyo Shirakawa, Jumpei Tokumaru, Mizue Ogi, Kenichiro Nishida, Takehiro Hirai, Kenta Shinozaki, Yoko Hamada, Hiroshi Kitagawa, Akihiko Horiguchi

**Affiliations:** ^1^ Department of Gastroenterology, Hepatobiliary and Pancreatic Medical Oncology Division Kanagawa Cancer Center Yokohama Japan; ^2^ Oncology Medical AstraZeneca K.K. Osaka Japan; ^3^ Evidence and Observational Research, Medical AstraZeneca K.K. Osaka Japan; ^4^ Department of Gastroenterological Surgery Fujita Health University School of Medicine Nagoya Japan

**Keywords:** antibiotics, chemotherapy, cholangiocarcinoma, cholangitis, drug therapy

## Abstract

**Purpose:**

To describe the real‐world treatment patterns of systemic therapies for biliary tract cancer (BTC) and to examine the frequency and management of biliary infection in Japan.

**Methods:**

Patients diagnosed with BTC and prescribed systemic therapy between January 2011 and September 2020 were retrieved from the Japanese Medical Data Vision database. The look‐back period was set to 5 years. Patient characteristics, treatment patterns, and biliary infection‐induced treatment interruption were analyzed.

**Results:**

The full analysis set comprised 22 742 patients with a mean age of 71.0 years and 61.6% were male. The most common BTC type was extrahepatic cholangiocarcinoma (44.6%). The three most common first‐line regimens were S‐1 monotherapy (33.0%), gemcitabine+cisplatin (32.5%), and gemcitabine monotherapy (18.7%) over the entire observation period (January 2011–September 2021). Patients who received monotherapies tended to be older. Biliary infection‐induced treatment interruption occurred in 29.5% of patients, with a median time to onset of 64.0 (interquartile range 29.0–145.0) days. The median duration of intravenous antibiotics was 12.0 (interquartile range 4.0–92.0) days.

**Conclusions:**

These results demonstrated potential challenges of BTC in Japanese clinical practice particularly use of multiple regimens, commonly monotherapies, which are not recommended as first‐line treatment, and the management of biliary infections during systemic therapy.

## Graphical abstract

This large‐scale database study provides a comprehensive overview of the systemic therapies for biliary tract cancer and biliary infections in real‐world clinical practice in Japan prior to the immunotherapy era. Ueno and colleagues identified some challenges related to multiple first‐line treatments and the management of biliary infections during systemic therapy.

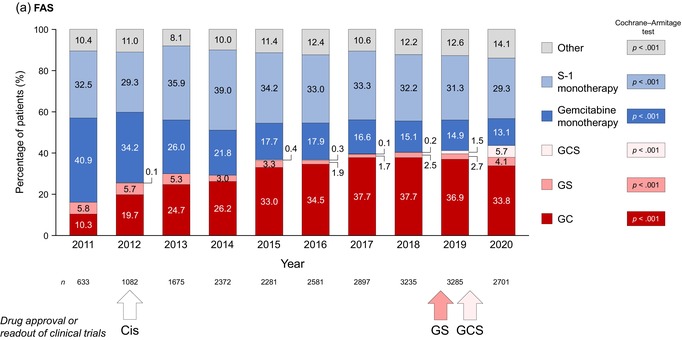

## 1 INTRODUCTION

Biliary tract cancers (BTC) occur in the intrahepatic or extrahepatic regions of the biliary tract, the gallbladder, and the ampulla of Vater (AoV). Although the incidence of BTC varies by geographical region and ethnicity, Japan has a relatively high incidence of 17.6 cases per 100 000 people annually for cholangiocarcinoma and gallbladder cancer (GBC).^1^ Patients with BTC are often diagnosed at an advanced stage and their survival is poor^2^; a 5‐year survival rate of 2.9% was reported for patients with metastatic cholangiocarcinoma or GBC in 2019.^1^

Despite gemcitabine+cisplatin (GC) being the global standard of care, other combination chemotherapies, such as gemcitabine+cisplatin+S‐1 (GCS) and gemcitabine+S‐1 (GS), have been considered as standard regimens for unresectable BTC in Japan.^3^ The 2019 Evidence‐based Clinical Guidelines for the Management of Biliary Tract Cancers recommended three combination chemotherapies (GC, GS, or GCS) equally as the standard first‐line chemotherapies for unresectable cases, albeit without clear guidance on which regimen to use.^4^

The clinical situation is also influenced by the emergence of immunotherapy combined with GC. Durvalumab+GC showed a significant clinical benefit on overall survival (OS) in patients with previously untreated, unresectable or metastatic BTC, or recurrent disease.^5^ Subsequently, pembrolizumab+GC was shown to prolonged OS in patients with unresectable BTC.^6^ These combination therapies are now recommended as preferred regimens in the National Comprehensive Cancer Network guidelines.^7^

Although these immunotherapies were studied in combination with GC, other regimens, including combination chemotherapies and monotherapies, are often used in clinical practice, and the recent real‐world treatment patterns for unresectable BTC in Japan have never been described in a large study population. With the emergence of novel treatment regimens, it is important to clarify the treatment patterns and to investigate why regimens other than the global standard of care were chosen in clinical practice.

Biliary infection and obstruction are other major challenges in the treatment for BTC, being common events and limiting systemic therapy.^8^, ^9^ Most patients with biliary obstruction require biliary drainage together with a delay or interruption to systemic therapy. Biliary infections, such as acute cholangitis, are sometimes associated with biliary obstruction and can worsen the patient's condition, in addition to necessitating treatment interruption, and cholangitis has been reported as a common cause of death in patients with advanced BTC.^10^ However, there are limited data regarding the incidence, number of hospitalizations, treatment duration, or clinical outcomes of biliary infections during cancer treatment in the real world. Thus, it is important to determine the frequency of biliary infection and the duration of its treatment to help understand its impact on the treatment of BTC in clinical practice.

Therefore, our objectives were to describe the real‐world patterns of systemic therapies for BTC and to examine the frequency and management of biliary infection during systemic therapy in Japan.

## 2 METHODS

### 2.1 Ethics

This study conformed to the ethical principles set forth in the Declaration of Helsinki, Good Clinical Practice, and Good Pharmacoepidemiology Practice, including the applicable legislation for noninterventional studies in Japan, which do not require ethical approval or informed consent for studies using data from a commercially available deidentified claims database. The study protocol was approved by an independent, nonprofit ethics committee (MINS Institutional Review Board; MINS‐REC‐220207; March 17, 2022).

### 2.2 Data source

We used inpatient and outpatient data from the Medical Data Vision (MDV) database (https://en.mdv.co.jp/), which comprises health claims data and administrative data or diagnosis procedure combination (DPC) data in Japan. It is one of the largest databases of its type, containing data for over 40 million patients treated across more than 460 hospitals in Japan (approximately 23% of all acute care hospitals in Japan), and it has been used in several hundred studies to date.^11^, ^12^, ^13^ The DPC system is a flat‐fee payment system used in Japan for reimbursement of patient care. In this system, diagnoses are recorded using International Classification of Disease (ICD) codes, treatments are recorded using Anatomical Therapeutic Classification codes, and procedures and tests are recorded using procedural codes. Owing to the data structure, the MDV database can be utilized for the purpose of obtaining real‐world evidence for specific clinical settings. Because all data are extracted in an anonymous manner and the institutions are not linked, patients cannot be tracked across multiple institutions.

### 2.3 Patients

We extracted data for patients who met the following eligibility criteria: diagnosis of BTC, prescription of a systemic therapy between January 2011 and September 2020, age ≥18 years at diagnosis, and at least one record after the index date. Follow‐up data were extracted through to the last available record or September 2021. The index date was defined as the date of the first recorded prescription of an eligible systemic therapy. To investigate the background characteristics and medical history, including prior malignancies or use of chemotherapy, data were collected for each patient for up to 5 years prior to the index date or from the start of the database in 2008.

### 2.4 Study endpoints

The primary endpoint was the proportion of patients who received each first‐line regimen. Secondary endpoints were patient and tumor characteristics according to the first‐line regimen, as well as the duration of first‐line systemic treatments. Exploratory endpoints included treatment interruption due to biliary infection, the onset of biliary infection, and the proportion of patients who received a second‐line treatment by tumor site. For post hoc analyses, additional exploratory endpoints included the types of first‐line regimens prescribed according to year and age‐group (<70 or ≥70 years).

We extracted the following data from the MDV database: patient/tumor characteristics (Table S1), systemic chemotherapy (Table S2), and surgical and biliary drainage procedures (Table S3). The start and end dates of first‐line systemic therapies are defined in Table S4. We also analyzed the endpoints related to biliary infection described in Table S5.

### 2.5 Data analyses

The full analysis set (FAS) comprised all patients who satisfied the study eligibility criteria. Because some patients potentially received systemic therapy as adjuvant therapy or to treat malignancies other than BTC, the advanced‐Tx subset was defined for post hoc analyses as a more appropriate population for the purpose of this study. This subset excluded patients who received systemic treatment within 12 weeks after surgery with a curative intent and patients who did not receive any systemic treatment within 3 years after diagnosis of BTC.

All data were analyzed descriptively in terms of the number and percentages of patients, or the mean and standard deviation (SD) or median and interquartile range (IQR), as appropriate. The time‐trends in proportions of patients receiving each first‐line regimen were assessed using the Cochrane–Armitage test (FAS only). Patient and tumor characteristics were compared among the first‐line regimens using one‐way analysis of variance for continuous variables and the *χ* ^2^ test for categorical variables (FAS only). A two‐sided significance level of .05 was used in all tests. All analyses were considered exploratory and were not adjusted for multiplicity. Missing data were not imputed. SAS Viya version 3.5 (SAS Institute, Cary, NC) was used for data analyses.

## 3 RESULTS

### 3.1 Patients

**FIGURE 1:**
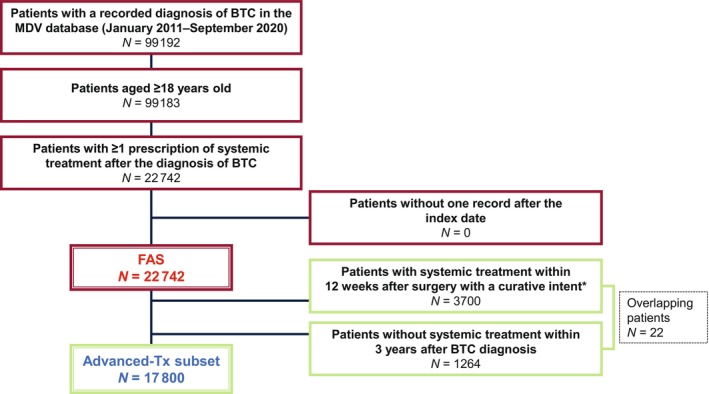
Patient disposition. The FAS may also include (1) patients who received systemic therapy as adjuvant treatment and/or (2) patients who received systemic therapy for malignancies other than BTC. Therefore, the advanced‐Tx subset excluded patients who received systemic treatment within 12 weeks after surgery with a curative intent and patients without systemic treatment within 3 years after BTC diagnosis. *Surgery with a curative intent included major hepatectomy, minor hepatectomy, pancreaticoduodenectomy, major hepatectomy and pancreaticoduodenectomy, and cholecystectomy. BTC, biliary tract cancer; FAS, full analysis set; MDV, Medical Data Vision.

Of 99 192 patients with a recorded diagnosis of BTC, 22 742 were included in the FAS. The advanced‐Tx subset used for post hoc analyses comprised 17 800 patients (Figure 1).

In the FAS, the mean ± SD age was 71.0 ± 9.3 years. Nearly two‐thirds (61.6%) of the patients were male (Table 1). Extrahepatic cholangiocarcinoma (EHCC) was the most common type of BTC (44.6%; cholangiocarcinoma, 33.8%; perihilar cholangiocarcinoma, 10.8%), followed by GBC (25.1%), intrahepatic cholangiocarcinoma (IHCC; 22.1%), and AoV cancer (7.5%). Almost one‐third (30.5%) of the patients had previously undergone surgical treatment for BTC and 2.8% underwent surgery after systemic treatment. Biliary drainage was performed in 39.8% of patients, primarily endoscopic biliary drainage/metallic stenting (31.1%). The patient and tumor characteristics of the advanced‐Tx subset were similar to those of the FAS, except for the proportion of patients who underwent surgery for BTC before systemic therapy, which was 17.6%.

**TABLE 1:** Patient characteristics.

Characteristic	FAS	Advanced‐Tx subset
*N*	22 742	17 800
Age, years, mean ± SD	71.0 ± 9.3	71.2 ± 9.4
Sex		
Female	8734 (38.4)	6897 (38.7)
Male	14 008 (61.6)	10 903 (61.3)
BMI, kg/m^2^, mean ± SD [*n*]	22.33 ± 3.66 [19 519]	22.33 ± 3.74 [15 028]
Tumor type/site		
IHCC	5017 (22.1)	4291 (24.1)
EHCC	10 151 (44.6)	7661 (43.0)
Cholangiocarcinoma	7694 (33.8)	5544 (31.1)
Perihilar cholangiocarcinoma	2457 (10.8)	2117 (11.9)
GBC	5701 (25.1)	4627 (26.0)
AoV	1707 (7.5)	1075 (6.0)
Unknown site	166 (0.7)	146 (0.8)
Comorbidities		
Cancer other than BTC	12 697 (55.8)	10 092 (56.7)
Non‐metastatic cancer	5180 (22.8)	3684 (20.7)
Metastatic cancer	7517 (33.1)	6408 (36.0)
Diabetes mellitus	9868 (43.4)	7085 (39.8)
Pancreaticobiliary maljunction	120 (0.5)	79 (0.4)
Primary sclerosing cholangitis	5805 (25.5)	4441 (24.9)
Intrahepatic gallstone	1507 (6.6)	1125 (6.3)
Cholelithiasis (gallstone, gallbladder stone)	2847 (12.5)	2065 (11.6)
Gallbladder polyp	313 (1.4)	199 (1.1)
Gallbladder adenomyomatosis	336 (1.5)	217 (1.2)
Obesity	68 (0.3)	47 (0.3)
Hyperlipidemia	4799 (21.1)	3629 (20.4)
Familial adenomatous polyposis	371 (1.6)	260 (1.5)
Surgery for BTC (before systemic therapy)	6930 (30.5)	3126 (17.6)
Surgery for BTC (after systemic therapy)	631 (2.8)	428 (2.4)
Biliary drainage^a^	9056 (39.8)	6833 (38.4)
PTBD	746 (3.3)	573 (3.2)
ENBD	1188 (5.2)	681 (3.8)
EUS‐BD	50 (0.2)	48 (0.3)
EBD/MS	7072 (31.1)	5531 (31.1)

*Note*: Values are *n* (%) unless specified otherwise.

Abbreviations: AoV, ampulla of Vater; BMI, body mass index; BTC, biliary tract cancer; EBD, endoscopic biliary drainage; EHCC, extrahepatic cholangiocarcinoma; ENBD, endoscopic nasobiliary drainage; EUS‐BD, endoscopic ultrasonography‐biliary drainage; FAS, full analysis set; GBC, gallbladder carcinoma; IHCC, intrahepatic cholangiocarcinoma; MS, metallic stenting; PTBD, percutaneous transhepatic biliary drainage; SD, standard deviation.

^a^ Two or more biliary drainage procedures were prioritized and counted as follows: PTBD+EBD/MS: PTBD; ENBD+EBD/MS: ENBD.

### 3.2 First‐line treatment

#### 3.2.1 Types of first‐line treatment regimens during the entire observation period

In the FAS, the two most common first‐line regimens were S‐1 monotherapy (33.0%) and GC (32.5%), followed by gemcitabine monotherapy (18.7%), GS (3.1%), GCS (1.0%), and “other” therapies (11.6%). In the advanced‐Tx subset, the three most common first‐line regimens were GC (38.3%), S‐1 monotherapy (25.7%), and gemcitabine monotherapy (20.2%).

#### 3.2.2 Patient and tumor characteristics according to first‐line treatment

The patient and tumor characteristics according to the first‐line regimen are summarized in Table 2 for the FAS and Table S6 for the advanced‐Tx subset.

**TABLE 2:** Characteristics of patients according to first‐line treatment (FAS).

Characteristic	All patients	GC	GS	GCS	Gemcitabine monotherapy	S‐1 monotherapy	Other	*p‐*value^a^
*N*	22 742	7394	715	229	4259	7510	2635	
Age, years, mean ± SD	71.0 ± 9.3	68.8 ± 8.8	68.6 ± 8.9	66.0 ± 9.8	73.2 ± 8.7	72.2 ± 9.2	71.6 ± 10.0	<0.001
Sex								
Female	8734 (38.4)	2884 (39.0)	298 (41.7)	79 (34.5)	1699 (39.9)	2829 (37.7)	945 (35.9)	0.002
Male	14 008 (61.6)	4510 (61.0)	417 (58.3)	150 (65.5)	2560 (60.1)	4681 (62.3)	1690 (64.1)	
BMI, kg/m^2^, mean ± SD [*n*]	22.33 ± 3.66 [19 519]	22.35 ± 3.50 [6714]	22.37 ± 7.14 [568]	22.86 ± 3.79 [204]	22.16 ± 3.55 [3500]	22.38 ± 3.42 [6198]	22.32 ± 3.60 [2335]	0.023
Tumor type/site								
IHCC	5017 (22.1)	2045 (27.7)	181 (25.3)	75 (32.8)	800 (18.8)	1125 (15.0)	791 (30.0)	<0.001
EHCC	10 151 (44.6)	2899 (39.2)	296 (41.4)	79 (34.5)	2145 (50.4)	3892 (51.8)	840 (31.9)	
Cholangiocarcinoma	7694 (33.8)	1892 (25.6)	231 (32.3)	39 (17.0)	1645 (38.6)	3187 (42.4)	700 (26.6)	
Perihilar cholangiocarcinoma	2457 (10.8)	1007 (13.6)	65 (9.1)	40 (17.5)	500 (11.7)	705 (9.4)	140 (5.3)	
GBC	5701 (25.1)	2100 (28.4)	196 (27.4)	67 (29.3)	995 (23.4)	1622 (21.6)	721 (27.4)	
AoV	1707 (7.5)	303 (4.1)	38 (5.3)	7 (3.1)	288 (6.8)	813 (10.8)	258 (9.8)	
Unknown site	166 (0.7)	47 (0.6)	4 (0.6)	1 (0.4)	31 (0.7)	58 (0.8)	25 (0.9)	
Surgery for BTC^b^ (before systemic therapy)	6930 (30.5)	1408 (19.0)	144 (20.1)	25 (10.9)	952 (22.4)	3713 (49.4)	688 (26.1)	<0.001
Minor hepatectomy	888 (3.9)	205 (2.8)	24 (3.4)	5 (2.2)	109 (2.6)	343 (4.6)	202 (7.7)	
Major hepatectomy	1196 (5.3)	254 (3.4)	23 (3.2)	2 (0.9)	165 (3.9)	634 (8.4)	118 (4.5)	
PD	3201 (14.1)	475 (6.4)	62 (8.7)	10 (4.4)	423 (9.9)	2087 (27.8)	144 (5.5)	
Major HPD	58 (0.3)	19 (0.3)	1 (0.1)	0	2 (0.0)	32 (0.4)	4 (0.2)	
Cholecystectomy	614 (2.7)	108 (1.5)	5 (0.7)	3 (1.3)	96 (2.3)	335 (4.5)	67 (2.5)	
Simple cholecystectomy	568 (2.5)	149 (2.0)	16 (2.2)	2 (0.9)	77 (1.8)	200 (2.7)	124 (4.7)	
Laparotomy	405 (1.8)	198 (2.7)	13 (1.8)	3 (1.3)	80 (1.9)	82 (1.1)	29 (1.1)	
Surgery for BTC^b^ (after systemic therapy)	631 (2.8)	209 (2.8)	27 (3.8)	26 (11.4)	82 (1.9)	141 (1.9)	146 (5.5)	<0.001
Biliary drainage	9056 (39.8)	3021 (40.9)	223 (31.2)	86 (37.6)	1784 (41.9)	3410 (45.4)	532 (20.2)	<0.001

*Note*: Values are *n* (%) unless specified otherwise.

Abbreviations: AoV, ampulla of Vater; BMI, body mass index; BTC, biliary tract cancer; EHCC, extrahepatic cholangiocarcinoma; FAS, full analysis set; GBC, gallbladder carcinoma; GC, gemcitabine+cisplatin; GCS, gemcitabine+cisplatin+S‐1; GS, gemcitabine+S‐1; HPD, hepatectomy and pancreaticoduodenectomy; IHCC, intrahepatic cholangiocarcinoma; PD, pancreaticoduodenectomy; SD, standard deviation.

^a^ One‐way analysis of variance for continuous variables and the *χ* ^2^ test for categorical variables.

^b^ Two or more procedures were prioritized and counted as follows: Minor hepatectomy+PD: PD; Minor hepatectomy+cholecystectomy: cholecystectomy; Minor hepatectomy+simple cholecystectomy: minor hepatectomy; Minor hepatectomy+laparotomy: laparotomy; Major hepatectomy+simple cholecystectomy: major hepatectomy; Major hepatectomy+laparotomy: major hepatectomy; PD+cholecystectomy: PD; PD+simple cholecystectomy: PD; Simple cholecystectomy+laparotomy: laparotomy; Cholecystectomy+simple cholecystectomy: cholecystectomy; Minor hepatectomy+simple cholecystectomy+laparotomy: laparotomy; Major hepatectomy+PD: HPD.

In the FAS, there were significant differences among the groups of patients for most of the baseline patient/tumor characteristics. The mean ages of patients prescribed gemcitabine monotherapy or S‐1 monotherapy were 73.2 ± 8.7 years and 72.2 ± 9.2 years, respectively. Among patients prescribed GC, GS, and GCS, the mean ages were 68.8 ± 8.8 years, 68.6 ± 8.9 years, and 66.0 ± 9.8 years, respectively. Approximately half of the patients prescribed gemcitabine (50.4%) or S‐1 (51.8%) monotherapies were diagnosed with EHCC. The proportions of cases of EHCC among patients prescribed GC, GS, or GCS were 39.2%, 41.4%, and 34.5%, respectively. There were also significant differences among the groups of patients regarding the frequencies of surgery for BTC before systemic therapy, surgery after systemic therapy, and biliary drainage in the FAS.

#### 3.2.3 Annual trends in first‐line treatment regimens

**FIGURE 2:**
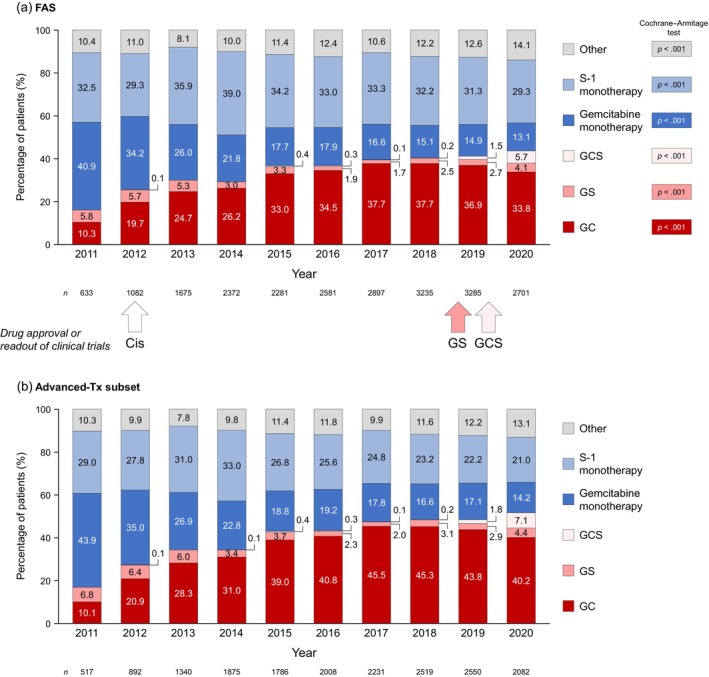
Annual transition of first‐line regimens in the FAS (a) and advanced‐Tx subset (b). The Cochrane–Armitage test was used to examine the significance of time‐trends for each therapy in the FAS; no statistical tests were performed for the advanced‐Tx subset. Cis, cisplatin; FAS, full analysis set; GC, gemcitabine+cisplatin; GCS, gemcitabine+cisplatin+S‐1; GS, gemcitabine+S‐1.

Figure 2a shows the annual distribution of first‐line treatment regimens in the FAS together with the approximate approval/readout of clinical trials for cisplatin, GS, and GCS. Over time, there were significant changes in the distribution of first‐line regimens (all *p* < 0.001). Prescriptions for gemcitabine monotherapy declined from 40.9% in 2011 to 13.1% in 2020, but S‐1 monotherapy remained relatively stable, at approximately 30%–35%, albeit with a peak of 39.0% in 2014. In 2020, gemcitabine or S‐1 as monotherapy accounted for over 40% of prescriptions. Prescriptions for GC increased from 10.3% in 2011 to 33.8% in 2020, peaking at 37.7% in 2017/2018. Prescriptions for GS remained low (<6%). GCS was prescribed sporadically prior to 2018 and increased to 5.7% in 2020.

In the advanced‐Tx subset (Figure 2b), the change in prescriptions for GC showed a similar trend to that in the FAS. Monotherapies were prescribed to small proportions of patients in 2020 (gemcitabine: 14.2%; S‐1: 21.0%).

**FIGURE 3:**
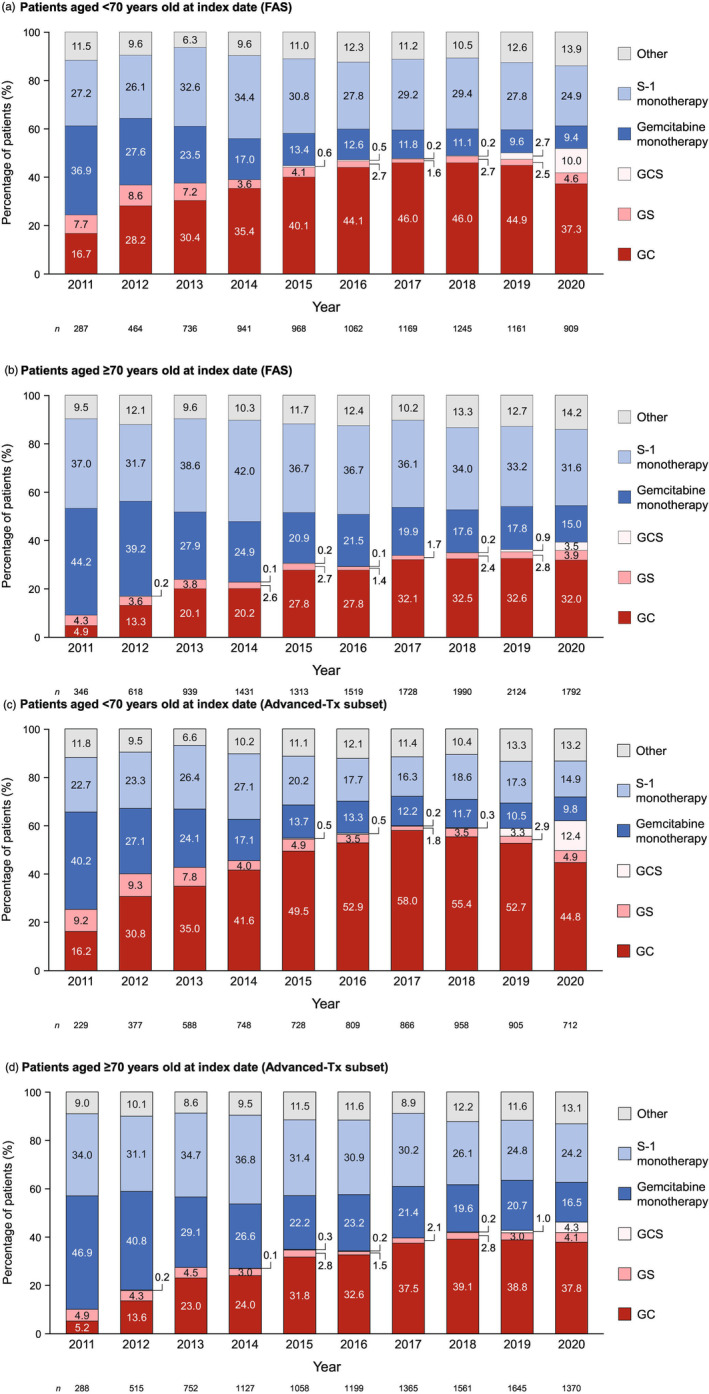
Annual transition of first‐line regimens among patients aged <70 years or ≥70 years at the index date in the FAS (a,b) and advanced‐Tx subset (c,d). FAS, full analysis set; GC, gemcitabine+cisplatin; GCS, gemcitabine+cisplatin+S‐1; GS, gemcitabine+S‐1.

In post hoc analyses, the annual trends in prescription practices among patients aged <70 or ≥70 years at the index date were assessed for the FAS and advanced‐Tx subset (Figure 3). Among patients aged <70 years in the FAS, prescriptions for GC reached a peak of 46.0% in 2017/2018 and decreased to 37.3% alongside a rapid increase of GCS prescriptions to 10.0% in 2020. Prescriptions for GC among patients aged ≥70 years were stable, at approximately 32% in 2017–2020. Throughout the study period, the proportion of patients prescribed monotherapies tended to be higher in patients aged ≥70 years than in patients aged <70 years.

Among patients aged <70 years in the advanced‐Tx subset, GC prescriptions reached a peak of 58.0% in 2017 and decreased to 44.8% alongside a rapid increase of GCS prescriptions to 12.4% in 2020. In patients aged ≥70 years, GC prescriptions peaked at 39.1% in 2018 and remained stable thereafter, whereas prescriptions for other combination therapies increased slightly, from 2.8% in 2018 to 4.1% in 2020 for GS and from 0.2% to 4.3% for GCS.

#### 3.2.4 Duration of first‐line treatment

The median duration of first‐line treatment was 99.0 days (IQR: 28.0–225.0 days) in the FAS (Table 3). Excluding “other” therapies, the median durations of first‐line treatment ranged from 71.0 days for GCS to 130.0 days for GS. In the advanced‐Tx subset, the median durations ranged from 71.0 days for GCS to 120.0 days for GS.

**TABLE 3:** Duration of first‐line treatment in patients with BTC (FAS and Advanced‐Tx subset).

	All patients	GC	GS	GCS	Gemcitabine monotherapy	S‐1 monotherapy	Other
FAS							
*N*	22 742	7394	715	229	4259	7510	2635
Duration, days	99.0 (28.0–225.0)	113.0 (43.0–225.0)	130.0 (50.0–274.0)	71.0 (29.0–141.0)	95.0 (29.0–228.0)	127.0 (29.0–260.0)	15.0 (1.0–117.0)
Advanced‐Tx subset							
*N*	17 800	6822	635	217	3597	4566	1963
Duration, days	85.0 (22.0–211.0)	108.0 (43.0–225.0)	120.0 (45.0–257.0)	71.0 (29.0–135.0)	85.0 (26.0–211.0)	85.0 (15.0–218.0)	10.0 (1.0–106.0)

*Note*: Values are median (interquartile range).

Abbreviations: BTC, biliary tract cancer; FAS; full analysis set; GC, gemcitabine+cisplatin; GCS, gemcitabine+cisplatin+S‐1; GS, gemcitabine+S‐1.

### 3.3 Second‐line treatment during the entire study period

Second‐line treatment regimens were documented for 9003 (39.6%) of 22 742 patients in the FAS and 7042 (39.6%) of 17 800 patients in the advanced‐Tx subset (Table S7). In the FAS, the frequency of second‐line treatment was 46.7%, 34.4%, 33.1%, 31.7%, and 20.1% among patients who received GC, gemcitabine monotherapy, S‐1 monotherapy, GS, and GCS as first‐line regimens, respectively. The most frequent second‐line regimen was S‐1 monotherapy, which was used in 4146 patients (Table S8).

### 3.4 Biliary infection during the entire study period

Treatment interruptions due to biliary infection were observed in 6712 (29.5%) of 22 742 patients in the FAS, with a median time to onset of 64.0 days (IQR: 29.0–145.0 days). The median duration of intravenous antibiotics treatment was 12.0 days (IQR: 4.0–92.0 days) (Table S9). In the FAS, of 6712 patients with biliary infection, 4844 (72.2%) were hospitalized at least once with a median duration (across one/more visits) of 21.5 days (IQR: 10.0–41.0 days). Among 16 030 patients without biliary infection, 8213 (51.2%) were hospitalized at least once with a median duration of 14.0 days (IQR: 7.0–25.0 days).

## 4 DISCUSSION

This large‐scale study of 22 742 patients with BTC illustrated the significant shifting trends in first‐line systemic therapy and the diversity of treatment regimens in Japanese real‐world settings, despite GC being the global standard of care. This study has demonstrated that treatment options for BTC outside those proposed in guidelines are used in clinical practice. This study also identified the factors associated with such treatments that should be addressed. The most frequent type of BTC in this study (in the FAS) was EHCC, which accounted for 44.6% of cases (cholangiocarcinoma: 33.8%; perihilar cholangiocarcinoma: 10.8%), followed by GBC (25.1%), IHCC (22.1%), and AoV cancer (7.5%). This distribution is quite similar to that reported in an analysis of 58 438 patients with malignant BTC registered in the National Cancer Registry, in which EHCC was the most frequent (24 602; 42.1%), followed by GBC (16 568; 28.4%), IHCC (12 497; 21.4%), and AoV cancer (4613; 7.9%).^14^

In terms of first‐line treatment, we found that the two most common regimens prescribed during the entire study period were S‐1 monotherapy (33.0%) and GC (32.5%). Over time, we observed significant variation in the prescriptions for first‐line regimens. In particular, prescriptions for GC increased substantially from 10.3% to 33.8% between 2011 and 2020, primarily in line with the decrease in gemcitabine monotherapy, which declined from 40.9% to 13.1% during the same period. GC was approved for BTC in 2012, and its prescriptions increased gradually to a peak in 2017. Although GS was used for BTC without established evidence, since 2017, the combinations GS and GCS have become treatment choices based on the results of randomized studies.^15^, ^16^ The annual trends in prescriptions for GC thus reflect the changes in treatment for BTC. We also determined the characteristics of patients according to their first‐line treatment, which revealed some clinically relevant differences, including age and primary tumor site. These findings suggest that physicians in Japan were considering relevant patient/tumor characteristics, when selecting first‐line treatment during the study period.

Although GC has been the standard of care for advanced BTC globally, less than half of the patients received GC in this study. This may be primarily due to concerns about the toxicities of cisplatin in elderly patients who are at high risk of renal dysfunction.^17^ In fact, this study showed that monotherapies tended to be chosen as first‐line treatment for elderly patients and their distributions remained stable in the last 5 years of the study period. Because BTC is mainly diagnosed in patients aged >70 years in Japan,^14^ concerns about toxicities may be associated with the decisions not to administer combination chemotherapies, including cisplatin, and the low use of GC, even in recent years. Other combinations, including GS and GCS, have been recommended as first‐line treatment in Japan, but they were prescribed to <10% of patients in this study, despite the increasing trend for GCS. The patient and disease characteristics were quite similar between the GC and GS groups, and it is difficult to speculate why GS was used in this study due to the lack of information on renal function. GS showed noninferior efficacy compared to GC in a clinical trial without concerns about renal dysfunction and requirement of hydration.^16^ The numerically longer median treatment duration for GS in this study (130.0 days for GS and 113.0 days for GC) might reflect the balanced effectiveness and toxicities of GS.

We found that GCS was sporadically used in Japan prior to 2019, but its use increased from 1.5% in 2019 to 5.7% in 2020. This seems understandable given the results of a large phase III trial, which showed that GCS was associated with a slightly longer OS (13.5 vs. 12.6 months, hazard ratio [HR] 0.79, 90% confidence interval [CI]: 0.628–0.996, *p* = 0.046) and progression‐free survival (7.4 vs. 5.5 months, HR 0.75, 95% CI: 0.577–0.970, *p* =0 .015) versus GC.^15^ When we consider patient‐related factors in this study, patients prescribed GCS tended to be younger, were more likely to have IHCC or perihilar cholangiocarcinoma, had a shorter treatment period, less frequently received subsequent systemic therapy, and more frequently underwent surgery after systemic therapy than patients prescribed other therapies. This implies that GCS is mainly reserved for younger patients who might be expected to tolerate it better. Furthermore, we think that GCS was frequently used in patients with unresectable disease who were potential candidates for conversion surgery or neoadjuvant treatment. Indeed, chemotherapy with the objective of subsequent surgical resection was a focus of numerous studies in patients with IHCC or perihilar cholangiocarcinoma.^18^ Supporting this approach, GCS was associated with a favorable objective response rate of up to 40% in a phase III study,^15^ which may contribute to subsequent resectability. In addition, shorter treatment periods might suggest the use of GCS for patients with more advanced BTC who were not expected to receive further treatment, even after considering GCS and all available drugs.

Gemcitabine and S‐1 were prescribed as monotherapies for first‐line treatment to more than 40% of patients in this study. Based on phase II trials of monotherapies for unresectable BTC, gemcitabine was approved in Japan for BTC in 2006 and S‐1 was approved in 2007.^19^, ^20^ S‐1 is an oral drug widely used in Japan that combines tegafur (a prodrug of 5‐fluorouracil) with two compounds (gimeracil and oteracil) to enhance the bioavailability of 5‐fluorouracil.^21^ In this study, monotherapies tended to be used in elderly patients and patients with EHCC. These decisions were likely based on the tolerability of combination chemotherapies, with consideration of the age‐related decline in renal function and the generally poor patient status due to more frequent biliary events in EHCC compared with other primary sites. Furthermore, oral medications might be more convenient than intravenous infusion, which could contribute to the more frequent use of S‐1 monotherapy.

In this study, the number of patients prescribed S‐1 monotherapy decreased from 7510 (33.0%) in the FAS to 4566 (25.7%) in the advanced‐Tx subset in post hoc analyses. The proportion of patients who underwent surgery prior to systemic therapy decreased from 49.4% to 23.7%, and the median treatment duration decreased from 127.0 days to 85.0 days between the two patient populations. Most of the excluded patients were thought to have received S‐1 monotherapy as adjuvant treatment. While adjuvant S‐1 monotherapy seemed to be commonly used in the study period, even without established evidence, it became the standard of care in Japan following the initial publication of the ASCOT study in 2022, which showed that adjuvant S‐1 monotherapy significantly improved OS versus observation for resected BTC.^22^ Even in the advanced‐Tx subset, which excluded patients who probably received adjuvant S‐1 treatment, patients prescribed S‐1 monotherapy accounted for the second‐largest group of patients. We should also consider the possibility that S‐1 monotherapy was potentially used as a second‐line treatment in some patients if their first‐line treatment was not recorded in the MDV database (e.g., first‐line treatment was done at a hospital not involved in the MDV database or the patient transferred hospitals between treatments). In addition to the large population with first‐line treatment, S‐1 monotherapy was the most frequent second‐line treatment regimen in this study. Thus, S‐1 was widely used in multiple settings in BTC and played an important role in Japanese clinical practice in the study period.

Another objective of this study was to assess the frequency of and duration of treatment for biliary infection during systemic therapy for BTC. Biliary infection is a notable disease‐related complication that is closely associated with biliary obstruction and biliary stents, and is a major limiting factor for cancer treatment.^23^ In this study, approximately 30% of patients experienced biliary infection that required intravenous antibiotics and treatment interruption. The median duration of intravenous antibiotics administration was 12 days, although this may underestimate the actual duration of antibiotics administration because we only assessed intravenous antibiotics; some patients were probably treated with oral antibiotics before and/or after intravenous antibiotics. Although the Tokyo Guidelines 2018 recommend intravenous antibiotics (4–7 days), after successful biliary drainage and control of the infection site when managing acute cholangitis,^24^ the management of acute cholangitis related to malignancies is not well established. Acute cholangitis in patients with malignancies is associated with high mortality, antibiotic resistance, and empiric therapy being ineffective against the causative microorganism.^25^, ^26^ In addition, since some previous studies have reported worse outcomes associated with antibiotics use in patients prescribed immunotherapy for various types of cancer,^27^, ^28^ it is necessary to consider the potential impact of antibiotics during treatment on the effectiveness of immunotherapy for BTC. Although antibiotics are essential for the management of biliary infections, it is increasingly important to ensure they are used appropriately in the immunotherapy era. Regarding the treatment duration, meta‐analyses have reported that administration of antibiotics for <7 days did not significantly affect the clinical outcomes compared with a longer course of antibiotics in patients with acute cholangitis.^29^, ^30^ However, the appropriate duration of antibiotics administration in patients with acute cholangitis related to malignancies has not been specifically studied. Further investigations are needed to establish the management of cancer‐related biliary infections in terms of drug resistance patterns in Japan, as well as the choice of antibiotics, treatment duration, and the impact on cancer treatment.

### 4.1 Limitations

Some limitations of this study include the type of data recorded in the MDV database (administrative claims rather than complete medical records) and the inability to merge data for individual patients treated at different hospitals. As the MDV database is not based on the entire medical records or charts, it lacks data on the treatment aims including adjuvant setting, outcomes, or clinical features (e.g., cancer stage and resectability). The FAS may also capture treatments for malignancies other than BTC since it did not apply a restriction to the interval between the diagnosis of BTC and the start of chemotherapy, or multiple cancers. Despite these limitations, the results of post hoc analyses in the Advanced‐Tx subset showed generally consistent treatment trends with those of the FAS. Furthermore, because the MDV database includes data from a quarter of DPC hospitals and half of the hub hospitals for cancer treatment in Japan, the results of this study may reflect the treatment practice for BTC in the real‐world setting.

## 5 CONCLUSIONS

This study provides a comprehensive overview of the systemic therapies for BTC and biliary infections during treatment in real‐world clinical practice in Japan prior to the immunotherapy era. We identified some themes to examine in future studies, such as the frequent use of multiple regimens, including monotherapies, which are not recommended as first‐line treatment, and the management of biliary infections during systemic therapy.

## AUTHOR CONTRIBUTIONS

Study conception: Makoto Ueno, Mizue Ogi, Kenichiro Nishida, Takehiro Hirai, Kenta Shinozaki, and Akihiko Horiguchi. Study design: Sachiyo Shirakawa, Mizue Ogi, Kenichiro Nishida, Takehiro Hirai, and Kenta Shinozaki. Data collection: Sachiyo Shirakawa, Jumpei Tokumaru, Mizue Ogi, and Kenichiro Nishida. Data analysis: Sachiyo Shirakawa, Jumpei Tokumaru, Mizue Ogi, Kenichiro Nishida, and Takehiro Hirai. Data interpretation: all authors. Manuscript writing, first draft: Sachiyo Shirakawa. Manuscript writing, critical review: all authors. Final approval: all authors. Accountability for accuracy and integrity: all authors.

## CONFLICT OF INTEREST STATEMENT

Makoto Ueno reported lecture fees and other fees exceeding 1 million yen for attending (presenting) conferences from Taiho Pharmaceutical; and research funding exceeding 1 million yen from Taiho Pharmaceutical, AstraZeneca, Merck Biopharma, MSD, Astellas Pharma, Eisai, Ono Pharmaceutical, Incyte, Chugai Pharmaceutical, DFP, Daiichi Sankyo, Novartis, Boehringer Ingelheim, and J‐pharma. Sachiyo Shirakawa, Jumpei Tokumaru, Mizue Ogi, Kenichiro Nishida, Takehiro Hirai, Kenta Shinozaki, Yoko Hamada, and Hiroshi Kitagawa reported being employees of AstraZeneca K.K. Akihiko Horiguchi reported no conflicts of interest.

## Supporting information


Tables S1–S9.

## Data Availability

Data used in this study cannot be shared with external researchers due to the terms of the research contract with Medical Data Vision. However, researchers may contact Medical Data Vision directly for all data requests (https://en.mdv.co.jp/).
