# Supplementary material for: Real‐world evidence of systemic treatment practices for biliary tract cancer in Japan: Results of a database study
Source: J Hepatobiliary Pancreat Sci. 2024 May 27;31(7):468–80. doi: 10.1002/jhbp.1418 (PMC11503459; doi:10.1002/jhbp.1418)
Supplement: Supplementary file 1 — Tables S1–S9. [file JHBP-31-468-s001.docx]

# Supporting Information

**Real-world evidence of systemic treatment practices for biliary tract cancer in Japan: results of a database study**

Makoto Ueno, Sachiyo Shirakawa, Jumpei Tokumaru, Mizue Ogi, Kenichiro Nishida, Takehiro Hirai, Kenta Shinozaki, Yoko Hamada, Hiroshi Kitagawa, and Akihiko Horiguchi

| **Contents** | **Page** |
| --- | --- |
| **TABLE S1** Biliary tract cancer | 2 |
| **TABLE S2** Definitions of the systemic treatment regimens | 3 |
| **TABLE S3** Surgical and biliary drainage procedures for biliary tract cancers | 4 |
| **TABLE S4** Definitions of the start and end of the first-line systemic treatment regimens | 5 |
| **TABLE S5** Definitions of biliary infection and antibiotic treatment | 7 |
| **TABLE S6** Patient characteristics according to first-line treatment regimen (Advanced-Tx subset) | 8 |
| **TABLE S7** Frequency of second-line treatment according to the first-line regimen | 11 |
| **TABLE S8** Second-line treatment regimens in the full analysis set (*n* = 22 742) | 12 |
| **TABLE S9** Biliary infection and antibiotic treatment in patients with BTC (FAS and Advanced-Tx subset) | 13 |

## Supplemental Tables

**TABLE S1** Biliary tract cancer

| **Tumor site** | **Diagnostic term** | **ICD-10 code** |
| --- | --- | --- |
| Intrahepatic cholangiocarcinoma (IHCC) | Intrahepatic cholangiocarcinoma | C22.1 |
| Extrahepatic cholangiocarcinoma (EHCC) | Cholangiocarcinoma | C24.0 |
|  | Perihilar cholangiocarcinoma | C24.8 |
| Gallbladder carcinoma (GBC) | Gallbladder carcinoma | C23 |
| Ampulla of Vater carcinoma (AoV) | Ampulla of Vater carcinoma | C24.1 |
| Biliary tract cancer (unknown site) | Biliary tract cancer (unknown site) | C24.9 |

ICD-10, International Classification of Diseases, 10^th^ revision

**TABLE S2** Definitions of the systemic treatment regimens

| **Regimen** | **General method** | **ATC codes** |
| --- | --- | --- |
| GC | Cisplatin plus gemcitabine administered on days 1 and 8, every 3 weeks. Gemcitabine monotherapy following GC was included in GC | L01BC05, L01XA01 |
| GS | Gemcitabine administered on days 1 and 8, S-1 administered orally twice daily on days 1–14, every 3 weeks. Gemcitabine monotherapy or S-1 monotherapy following GS were included in GS | L01BC05, L01BC53 |
| GCS | Gemcitabine plus cisplatin administered on day 1, S-1 administered orally once daily on days 1–7, every 2 weeks. GS, gemcitabine monotherapy, or S-1 monotherapy following GCS were included in GCS | L01BC05, L01XA01, L01BC53 |
| Gemcitabine monotherapy | Gemcitabine administered on   1. Days 1, 8, and 15, every 4 weeks 2. Days 1 and 8, every 3 weeks 3. Day 1 every 2 weeks | L01BC05 |
| S-1 monotherapy | S1 administered orally twice daily on days 1–28, every 6 weeks | L01BC53 |
| Pembrolizumab | Pembrolizumab administered on day 1, every 3 or 6 weeks | L01XC18 |
| Pemigatinib | Pemigatinib administered orally once daily on days 1–14, every 3 weeks | L01EX20 |
| Other | Systemic treatments other than those listed above | L01 (other than those listed above) |

ATC, Anatomical Therapeutic Chemical classification; GC, gemcitabine+cisplatin; GCS, gemcitabine+cisplatin+S-1; GS, gemcitabine+S-1

**TABLE S3** Surgical and biliary drainage procedures for biliary tract cancers

| **Procedure** | **K code** |
| --- | --- |
| **Surgical procedures** |  |
| Minor hepatectomy | K6752, K6951, K6952, K6953, K6954, K695-21, K695-22, K695-23, K695-24 |
| Major hepatectomy | K6753, K677-21 (except K677-21+K7031), K677-22 (except K677-22+K7031), K6955, K6956, K6957, K695-25, K695-26 |
| Pancreaticoduodenectomy (PD) | K6754, K6772, K6773, K703 |
| Major hepatectomy and pancreaticoduodenectomy (HPD) | K6766, K6771, K677-21+K7031, K677-22+K7031 |
| Cholecystectomy | K6751 |
| Simple cholecystectomy | K672, K672-2 |
| Laparotomy | K636, K636-3 |
| **Biliary drainage procedures** |  |
| Percutaneous transhepatic biliary drainage (PTBD) | K682-2, K689 |
| Endoscopic nasobiliary drainage (ENBD) | K682-3 |
| Endoscopic ultrasonography-biliary drainage (EUS-BD) | K682-4 |
| Endoscopic biliary drainage (EBD)/metallic stenting (MS) | K688 |

**TABLE S4** Definitions of the start and end of the first-line systemic treatment regimens

| **Regimen** | **Start** | **End ^a^** |
| --- | --- | --- |
| GC | Simultaneous prescription of gemcitabine and cisplatin, but not S-1, on the index date | a) The last prescription of gemcitabine in the database  *or*  b) The last prescription of these drugs prior to the first prescription of an antineoplastic agent other than gemcitabine or cisplatin |
| GS | Simultaneous prescription of gemcitabine and S-1, but not cisplatin, on the index date | a) The last prescription of gemcitabine or S-1 in the database  *or*  b) The last prescription of these drugs prior to the first prescription of an antineoplastic agent other than gemcitabine or S-1 |
| GCS | Simultaneous prescription of gemcitabine, cisplatin, and S-1 on the index date | a) The last prescription of gemcitabine, cisplatin, or S-1 in the database  *or*  b) The last prescription of these drugs prior to the first prescription of an antineoplastic agent other than gemcitabine, cisplatin, or S-1 |
| Gemcitabine monotherapy | Prescription of gemcitabine but not any other antineoplastic agent on the index date | a) The last prescription of gemcitabine in the database  *or*  b) The last prescription of these drugs prior to the first prescription of an antineoplastic agent other than gemcitabine |
| S-1 monotherapy | Prescription of S-1 but not any other antineoplastic agent on the index date | a) The last prescription of S-1 in the database  *or*  b) The last prescription of these drugs prior to the first prescription of an antineoplastic agent other than S-1 |
| Other | Prescription of any antineoplastic agent other than gemcitabine, cisplatin, or S-1 on the index date | a) The last prescription of the drug prescribed on the index date in the database  *or*  b) The last prescription of these drugs prior to the first prescription of an antineoplastic agent other than the drug prescribed on the index date |

GC, gemcitabine+cisplatin; GCS, gemcitabine+cisplatin+S-1; GS, gemcitabine+S-1

^a^ Whichever came first

**TABLE S5** Definitions of biliary infection and antibiotic treatment

| **Endpoint** | **Definition** |
| --- | --- |
| First treatment interruption due to BI | Events that satisfied the following three criteria:  (a) no prescription of systemic treatment for BTC for ≥22 days  (b) onset of biliary infection (ICD-10 code K380) after starting first-line systemic treatment and before starting second-line treatment  (c) a+b occurred for the first time during the first-line treatment regimen |
| Start date of BI | Time within 1 week before and after and in the same month of diagnosis of biliary infection (ICD-10: K830) and starting an injectable antibiotic (ATC code: J01; drug usage code: 4. Injections) during first-line systemic treatment |
| End date of BI | The end date was defined as the last date of the last prescription of the antibiotic before restarting systemic treatment |
| BI | Combination of ICD-10 code K830 and administration of antibiotics (ATC code J01) via injection (drug usage code: 4. Injections) during first-line systemic treatment |
| Time to onset of BI | Number of days from the index date to the day of biliary infection onset |
| Time of treatment interruption | Day of biliary infection onset |

ATC, Anatomical Therapeutic Chemical classification; BI, biliary infection; BTC, biliary tract cancer; ICD-10, International Classification of Diseases, 10^th^ revision

**TABLE S6** Patient characteristics according to first-line treatment regimen (Advanced-Tx subset)

| **Characteristic** | **All patients** | **GC** | **GS** | **GCS** | **Gemcitabine monotherapy** | **S-1 monotherapy** | **Other** |
| --- | --- | --- | --- | --- | --- | --- | --- |
| *N* | 17 800 | 6822 | 635 | 217 | 3597 | 4566 | 1963 |
| Age, years, mean ± SD | 71.2 ± 9.4 | 68.8 ± 8.9 | 68.5 ± 8.9 | 66.0 ± 9.7 | 73.6 ± 8.8 | 73.4 ± 9.3 | 71.2 ± 10.1 |
| Sex |  |  |  |  |  |  |  |
| Female | 6897 (38.7) | 2678 (39.3) | 274 (43.1) | 77 (35.5) | 1446 (40.2) | 1715 (37.6) | 707 (36.0) |
| Male | 10 903 (61.3) | 4144 (60.7) | 361 (56.9) | 140 (64.5) | 2151 (59.8) | 2851 (62.4) | 1256 (64.0) |
| BMI, kg/m^2^, mean ± SD [*n*] | 22.33 ± 3.74 [15 028] | 22.38 ± 3.51 [6205] | 22.40 ± 7.48 [505] | 22.86 ± 3.84 [194] | 22.20 ± 3.60 [2935] | 22.31 ± 3.51 [3438] | 22.33 ± 3.62 [1751] |
| Tumor type/site |  |  |  |  |  |  |  |
| IHCC | 4291 (24.1) | 1931 (28.3) | 169 (26.6) | 71 (32.7) | 710 (19.7) | 788 (17.3) | 622 (31.7) |
| EHCC | 7661 (43.0) | 2621 (38.4) | 254 (40.0) | 74 (34.1) | 1790 (49.8) | 2286 (50.1) | 636 (32.4) |
| Cholangiocarcinoma | 5544 (31.1) | 1672 (24.5) | 195 (30.7) | 36 (16.6) | 1344 (37.4) | 1769 (38.7) | 528 (26.9) |
| Perihilar cholangiocarcinoma | 2117 (11.9) | 949 (13.9) | 59 (9.3) | 38 (17.5) | 446 (12.4) | 517 (11.3) | 108 (5.5) |
| GBC | 4627 (26.0) | 1969 (28.9) | 180 (28.3) | 64 (29.5) | 854 (23.7) | 1048 (23.0) | 512 (26.1) |
| AoV | 1075 (6.0) | 254 (3.7) | 28 (4.4) | 7 (3.2) | 216 (6.0) | 397 (8.7) | 173 (8.8) |
| Unknown site | 146 (0.8) | 47 (0.7) | 4 (0.6) | 1 (0.5) | 27 (0.8) | 47 (1.0) | 20 (1.0) |
| Surgery for BTC* (before systemic therapy) | 3126 (17.6) | 1007 (14.8) | 90 (14.2) | 18 (8.3) | 498 (13.8) | 1082 (23.7) | 431 (22.0) |
| Minor hepatectomy | 372 (2.1) | 107 (1.6) | 10 (1.6) | 1 (0.5) | 40 (1.1) | 96 (2.1) | 118 (6.0) |
| Major hepatectomy | 507 (2.8) | 165 (2.4) | 11 (1.7) | 1 (0.5) | 80 (2.2) | 181 (4.0) | 69 (3.5) |
| PD | 1122 (6.3) | 328 (4.8) | 38 (6.0) | 9 (4.1) | 197 (5.5) | 467 (10.2) | 83 (4.2) |
| HPD | 30 (0.2) | 14 (0.2) | 1 (0.2) | 0 | 1 (0.0) | 11 (0.2) | 3 (0.2) |
| Cholecystectomy | 167 (0.9) | 55 (0.8) | 2 (0.3) | 2 (0.9) | 26 (0.7) | 58 (1.3) | 24 (1.2) |
| Simple cholecystectomy | 548 (3.1) | 148 (2.2) | 16 (2.5) | 2 (0.9) | 76 (2.1) | 196 (4.3) | 110 (5.6) |
| Laparotomy | 380 (2.1) | 190 (2.8) | 12 (1.9) | 3 (1.4) | 78 (2.2) | 73 (1.6) | 24 (1.2) |
| Surgery for BTC ^a^ (after systemic therapy) | 428 (2.4) | 164 (2.4) | 23 (3.6) | 24 (11.1) | 47 (1.3) | 72 (1.6) | 98 (5.0) |
| Biliary drainage | 6833 (38.4) | 2797 (41.0) | 201 (31.7) | 81 (37.3) | 1513 (42.1) | 1801 (39.4) | 440 (22.4) |

*Note:* Values are *n* (%) unless specified otherwise

AoV, ampulla of Vater; BMI, body mass index; BTC, biliary tract cancer; EHCC, extrahepatic cholangiocarcinoma; GBC, gallbladder carcinoma; GC, gemcitabine+cisplatin; GCS, gemcitabine+cisplatin+S-1; GS, gemcitabine+S-1; HPD, hepatectomy and pancreaticoduodenectomy; IHCC, intrahepatic cholangiocarcinoma; PD, pancreaticoduodenectomy; SD, standard deviation

^a^ Two or more procedures were prioritized and counted as follows:

- Minor hepatectomy+PD: PD
- Minor hepatectomy+cholecystectomy: cholecystectomy
- Minor hepatectomy+simple cholecystectomy: minor hepatectomy
- Minor hepatectomy+laparotomy: laparotomy
- Major hepatectomy+simple cholecystectomy: major hepatectomy
- Major hepatectomy+laparotomy: major hepatectomy
- PD+cholecystectomy: PD
- PD+simple cholecystectomy: PD
- Simple cholecystectomy+laparotomy: laparotomy
- Cholecystectomy+simple cholecystectomy: cholecystectomy
- Minor hepatectomy+simple cholecystectomy+laparotomy: laparotomy
- Major hepatectomy+PD: HPD

**TABLE S7** Frequency of second-line treatment according to the first-line regimen

|  | **All patients** | **GC** | **GS** | **GCS** | **Gemcitabine monotherapy** | **S-1 monotherapy** | **Other** |
| --- | --- | --- | --- | --- | --- | --- | --- |
| FAS |  |  |  |  |  |  |  |
| *N* | 22 742 | 7394 | 715 | 229 | 4259 | 7510 | 2635 |
| Second-line, *n* (%) | 9003 (39.6) | 3454 (46.7) | 227 (31.7) | 46 (20.1) | 1464 (34.4) | 2487 (33.1) | 1325 (50.3) |
| Advanced-Tx subset |  |  |  |  |  |  |  |
| *N* | 17 800 | 6822 | 635 | 217 | 3597 | 4566 | 1963 |
| Second-line, *n* (%) | 7042 (39.6) | 3175 (46.5) | 209 (32.9) | 41 (18.9) | 1197 (33.3) | 1386 (30.4) | 1034 (52.7) |

FAS, full analysis set; GC, gemcitabine+cisplatin; GCS, gemcitabine+cisplatin+S-1; GS, gemcitabine+S-1

**TABLE S8** Second-line treatment regimens in the full analysis set (*n*=22 742)

|  | **Second-line regimen** | | | | | | |
| --- | --- | --- | --- | --- | --- | --- | --- |
|  | **All** | **GC** | **GS** | **GCS** | **Gemcitabine monotherapy** | **S-1 monotherapy** | **Other** |
| Number of patients who received second-line treatment | 9003 (39.6) | 1142 | 461 | 35 | 1337 | 4146 | 1882 |
| Tumor type/site |  |  |  |  |  |  |  |
| IHCC | 2179 (24.2) | 248 (21.7) | 138 (29.9) | 15 (42.9) | 189 (14.1) | 1062 (25.6) | 527 (28.0) |
| EHCC | 3849 (42.8) | 519 (45.4) | 161 (34.9) | 11 (31.4) | 760 (56.8) | 1742 (42.0) | 656 (34.9) |
| Cholangiocarcinoma | 2940 (32.7) | 406 (35.6) | 105 (22.8) | 7 (20.0) | 636 (47.6) | 1269 (30.6) | 517 (27.5) |
| Perihilar cholangiocarcinoma | 909 (10.1) | 113 (9.9) | 56 (12.1) | 4 (11.4) | 124 (9.3) | 473 (11.4) | 139 (7.4) |
| GBC | 2175 (24.2) | 251 (22.0) | 143 (31.0) | 8 (22.9) | 243 (18.2) | 1064 (25.7) | 466 (24.8) |
| AoV | 733 (8.1) | 119 (10.4) | 16 (3.5) | 1 (2.9) | 141 (10.5) | 249 (6.0) | 207 (11.0) |
| Unknown site | 67 (0.7) | 5 (0.4) | 3 (0.7) | 0 (0.0) | 4 (0.3) | 29 (0.7) | 26 (1.4) |

*Note:* Values are *n* (%) or *n*

AoV, ampulla of Vater; EHCC, extrahepatic cholangiocarcinoma; GBC, gallbladder carcinoma; GC, gemcitabine+cisplatin; GCS, gemcitabine+cisplatin+S-1; GS, gemcitabine+S-1; IHCC, intrahepatic cholangiocarcinoma

**TABLE S9** Biliary infection and antibiotic treatment in patients with BTC (FAS and Advanced-Tx subset)

|  | **All patients** | **GC** | **GS** | **GCS** | **Gemcitabine monotherapy** | **S-1 monotherapy** | **Other** |
| --- | --- | --- | --- | --- | --- | --- | --- |
| FAS |  |  |  |  |  |  |  |
| *N* | 22 742 | 7394 | 715 | 229 | 4259 | 7510 | 2635 |
| BI ^a^, *n* (%) | 6712 (29.5) | 2735 (37.0) | 287 (40.1) | 60 (26.2) | 1536 (36.1) | 1586 (21.1) | 508 (19.3) |
| Time to BI onset, days | 64.0  (29.0–145.0) | 58.0  (27.0–129.0) | 64.0  (31.0–143.0) | 57.0  (26.0–81.5) | 64.5  (30.0–144.0) | 78.0  (35.0–179.0) | 50.0  (21.0–134.0) |
| Duration of antibiotics, days | 12.0  (4.0–92.0) | 15.0  (5.0–103.0) | 16.0  (5.0–117.0) | 10.0  (3.5–70.0) | 14.0  (5.0–104.0) | 9.0  (4.0–63.0) | 9.0  (4.0–81.0) |
| Advanced-Tx subset |  |  |  |  |  |  |  |
| *N* | 17 800 | 6822 | 635 | 217 | 3597 | 4566 | 1963 |
| BI ^a^, *n* (%) | 5772 (32.4) | 2582 (37.8) | 264 (41.6) | 58 (26.7) | 1354 (37.6) | 1132 (24.8) | 382 (19.5) |
| Time to BI onset, days | 60.0  (28.0–134.0) | 57.0  (26.0–127.0) | 63.5  (30.5–135.5) | 57.0  (25.0–82.0) | 61.0  (29.0–132.0) | 71.0  (32.0–164.0) | 49.0  (20.0–116.0) |
| Duration of antibiotics, days | 14.0  (5.0–97.0) | 17.0  (5.0–106.0) | 19.0  (5.0–119.5) | 10.0  (3.0–61.0) | 15.0  (5.0–103.0) | 10.0  (4.0–73.0) | 9.0  (4.0–75.0) |

*Note:* Values are median (interquartile range) unless otherwise specified

BI, biliary infection; BTC, biliary tract cancer; FAS; full analysis set; GC, gemcitabine+cisplatin; GCS, gemcitabine+cisplatin+S-1; GS, gemcitabine+S-1

^a^ Patients starting antibiotic treatment
